# Quality of Life in Patients with Implantable Cardioverter Defibrillators

**Published:** 2006-07-01

**Authors:** Johnson Francis, Beena Johnson, Michael Niehaus

**Affiliations:** * Associate Professor of Cardiology, Medical College Calicut, Kerala, India; † Consultant in Psychological Medicine, Baby Memorial Hospital, Calicut, Kerala, India; ‡ Professor of Cardiology, Dept. of Cardiology and Angiology, Hannover Medical School, Carl-Neuberg-Str. 1, 30625 Hannover, Germany

**Keywords:** Implantable defibrillator, meta analysis, quality of life

## Abstract

**Background:**

The implantable cardioverter defibrillator (ICD) is a life saving device for individuals with life threatening ventricular arrhythmias. There is no doubt that it is a cost effective therapy in various congenital and acquired arrhythmogenic disorders. Nevertheless, shock delivery may be painful and frightening which causes psychological distress and deterioration of perceived quality of life.

**Methods:**

A systematic meta-analysis on studies reporting quality of life in patients implanted with ICDs was done using professional databases. Related articles and references of the relevant articles were also searched for suitable studies.

**Results:**

Thirty studies with a total of 3412 patients on implantable defibrillators were identified. Five of them were large randomised studies with a total of 1680 patients, while 25 were non-randomised studies. Medical Outcome Study 36-item Short Form health survey (SF -94 36) was the most common instrument used for assessment of quality of life. Only one of the 5 major randomised trial reported worsening of quality of life after implantation of a defibrillator. In the subgroup of patients receiving shocks, three out of the five trials reported worsening of quality of life.

**Summary:**

Most of the randomised studies showed either neutral or better quality of life in patients on implantable defibrillators. In the subset of patients receiving shocks, worsening of quality of life was found in most randomised studies. Therefore, activation of antitachycardia pacing should be performed in every ICD-patient in order to miminze painful shocks and consequent deterioration of quality of life.

## Introduction

Implantable cardioverter - defibrillators (ICD) have proven their value as life saving devices [1-3]. As the number of patients implanted with these devices rises world-wide, concerns about the quality of the life prolonged by the ICDs become more and more relevant. Patients on ICD are prone for deterioration of quality of life (QOL) due to worsening of the pre-existing cardiac disorder as they survive longer with the support of the device. Unexpected and often painful shocks can either be perceived as instances of life regained or as potential threats to survival by different patients. Systematic attempts at evaluation of QOL in ICD recipients have been there over the past decade. This paper aims at a comprehensive analysis of the literature on QOL in ICD recipients.

## Methods

A systematic accumulation of available data on QOL in patients with ICDs. PubMed searches were conducted using the search words implantable defibrillator, ICD, quality of life and QOL. The retrieved results were checked to identify relevant studies. Further studies were sought by searching for the related articles and the references of the retrieved article. Reports of major ICD trials were screened for data on quality of life.

## Results

Thirty studies on patients with ICDs reported data on QOL (Table 1) [4-33]. Of the total 4653 patients in these 30 studies, 3412 had ICDs implanted. The earliest study in 1993 reported on 57 patients [4. The largest available data is from the PainFREE Rx II trial with 634 patients on ICD [33]. The next largest study was the Antiarrhythmics Versus Implantable Defibrillators (AVID) trial with 416 patients on ICD with an equal number on amiodarone for comparison [1-23]. 22 studies had 50 or more patients on ICD while only seven had over hundred patients on ICD. Fourteen studies were published in the last decade while sixteen were in the current decade. Twelve studies used Medical Outcome Study 36-item Short Form health survey (SF - 36) for assessment of quality of life.

## Instruments for Assessment of QOL

SF - 36 was the most common instrument used for QOL assessment across the studies. It comprises of a 36-item short-form questionnaire constructed to survey health status in the Medical Outcomes Study [33].  SF - 36 is a generic instrument and not disease specific. There are eight multi-item scales, four each for physical and mental health assessment. The physical scales are: 1) Physical functioning, 2) Role physical, 3) Bodily pain and 4) General health. The scales for assessing mental health are: 1) Vitality, 2) Social functioning, 3) Role emotional and 4) Mental health.

Various other scales have also been used in the studies, like Ferrans and Powers Quality of Life Index [7,18,30], Quality-of-life Profile for the Chronically Ill [13], Quality of Life Index: Cardiac Version [1] and specifically developed quality of life questionnaire for ICD patients [10]. The latter two can be considered as disease specific measurements of QOL.

## Major Randomized Trials

Of the thirty studies available, five were large randomized trials (Table 2) with 1680 patients on ICD altogether. Three of them were primary prevention trials [17,27,33] while two of them were secondary prevention trials [21,23]. One primary prevention trial reported worsening of QOL after ICD while another reported neutral results and the third and most recent one reported improvement in QOL. This disparity was present among the secondary prevention trials as well, where one reported improvement while another reported worsening of QOL. Disparity was less between the trials in those patients receiving ICD shocks. Three trials reported worsening of QOL while one each reported neutral and better QOL in patients receiving shocks.

## Summary of QOL Data from Major Randomized Trials

### A] Primary Prevention Trials

#### 1. CABG Patch Trial

Patients with impaired left ventricular function and positive signal averaged electrocardiogram undergoing CABG were randomly assigned to ICD versus no antiarrhythmic treatment in the CABG Patch trial [17]. All ICD-patients were implanted with patch electrodes during thoracotomy for CABG application. SF-36 was used to evaluate the QOL in 490 (68%) of the 719 patients available at 6 month follow up. Of these, 262 patients had ICD while 228 were controls. Patients in the ICD group had lower levels of psychological well being than the control group, especially those who experienced shock delivery of the ICD. The study was terminated when statistical analysis suggested that there was no significant difference in the primary outcome of total mortality. The lack of effect of ICDs in reducing mortality has been attributed to better survival in patients who have undergone revascularisation by CABG.

#### 2. AMIOVIRT Trial

Amiodarone versus implantable cardioverter-defibrillator (AMIOVIRT) trial included one hundred three patients with non-ischemic dilated cardiomyopathy (NIDCM), left ventricular ejection fraction < or = 0.35, and asymptomatic non sustained ventricular tachycardia (NSVT) [27]. They were randomized to receive either amiodarone or an ICD. The authors described no statistically significant survival advantage after one year or three years. QOL was also similar in both treatment groups. This trial was also neutral towards change of QOL in those receiving ICD shocks. Quality of Well Being Schedule and the State Trait Anxiety Inventory were the instruments used for assessment in this study.

#### 3. PainFREE Rx II Trial

Pacing Fast Ventricular Tachycardia Reduces Shock Therapies (PainFREE Rx II) trial was the largest trial reporting data on QOL as well as the latest [33]. This trial compared the safety and utility of empirical anti-tachycardia pacing (ATP) with shocks for fast ventricular tachycardia in patients receiving ICD. Of 634 ICD patients 313 were randomized to empirical ATP while 321 patients received shocks as the initial therapy of spontaneous fast ventricular tachycardia. ATP was effective in 229 of 284 episodes in the ATP arm. QOL was measured with the SF-36 and found to be improved in patients with fast ventricular tachycardia in both arms but even more in the ATP arm. Of the 131 patients with fast ventricular tachycardia, 98 completed SF-36 both at baseline and at 1 year. The ATP arm had significant improvement in 5 subscales - physical functioning, role physical, bodily pain, social functioning and role emotional, while the shock arm had improvement in only bodily pain score. None of the subscale measurements were significantly reduced at 1 year in either arm.

### B] Secondary Prevention Trials

#### 1. The Canadian Implantable Defibrillator Study (CIDS)

CIDS [21] compared antiarrhythmic therapy versus ICD in patients resuscitated from cardiac arrest, and those with sustained ventricular tachycardia with syncope or ventricular tachycardia of more than 150 beats per minute with near syncope and left ventricular ejection fraction < or = 0.35 or syncope with inducible ventricular tachycardia.

The total mortality was lower in the ICD group, but the difference did not reach statistical significance. (P=0.09). QOL evaluation was done as a secondary endpoint in the study with the Rand Corporation's 38-item Mental Health Inventory (MHI) and the Nottingham Health Profile (NHP) for 317 English-speaking participants.  Perceived QOL was found to be improved more in the ICD group. Significant improvement from baseline to the 6- and 12-month follow-up assessment was seen for 7 of the 10 variables assessed. Quality of life did not improve in those patients who received > or =5 shocks from their device. Still they were comparable to the antiarrhythmic group in QOL. There was no difference between those who received no shock and those who received 1-4 shocks from their ICD. None of the quality-of-life variables improved with time in the antiarrhythmic therapy group.

#### 2. The Antiarrhythmics Versus Implantable Defibrillator (AVID) Trial 

AVID Trial [1] compared antiarrhythmic drug therapy with ICD in survivors of cardiac arrest and those with sustained ventricular tachycardia with syncope, or hemodynamically unstable sustained ventricular tachycardia with ejection fraction < or = 40%. The study was prematurely terminated when a statistically significant reduction in mortality was demonstrated in the ICD group. Perceived QOL was evaluated as a secondary endpoint using SF-36 and Quality of Life Index: Cardiac Version. Of the 800 eligible patients with a 1-year survival, 416 patients were in the ICD group and 384 patients in the antiarrhythmic group. Perceived QOL related was low in both groups, related to adverse symptoms of the particular therapy. The occurrence of one or more shocks was associated with significant reduction in QOL in the ICD group.

## Data from non-randomised studies

Of the 30 studies evaluated in this report, 25 were non-randomised (Table 3). Sixteen of these trials had 50 or more patients on ICD. Five of these trials reported improvement in QOL in ICD patients while 6 reported neutral results and five reported worsening of QOL. IICD implantation did not worsen QOL in recipients.

The earliest study which reported QOL data from ICD patients was by Luderitz et al [4]. Fifty-five of their 57 patients stated that it was worth having an ICD implanted. This study also documented that patients receiving more than 5 shocks had a higher anxiety level. The latest of the non-randomised studies was by Sears et al [32]. They noted that patients with high positive health expectations and high optimism reported better mental health and social functioning at follow-up assessment. These two factors may be targeted in future studies to improve QOL in ICD recipients.

The largest of the non-randomised studies actually reported on dual chamber defibrillators for restoration of sinus rhythm in atrial tachyarrhythmias. This study by Newman et al [25] had 267 patients implanted with DDD ICD. A total of 150 patients completed SF-36 evaluation. Significant improvement was noted in role-physical, physical functioning, vitality, mental health and social functioning scales. No deterioration of QOL was noted in those who received shocks.

In the next largest non-randomized study by Kamphuis et al [22] 133 patients were on ICD and 35 on other modalities of treatment. No significant difference in QOL was noted between the two groups. Physical function, mental health and social function improved with time in both treatment groups. In contrast, Stankoweit et al [15] who studied 132 patients on ICD noted reduced well being in 61% of them.

## Conclusions

Distressing aspects of ICD shocks are lack of warning, multiple shocks, and progressively increased sensations with multiple shocks. Three of the major randomised studies reported worsening of QOL in those patients receiving ICD shocks, especially when  patients were receiving five or more shocks. Despite the AMIOVIRT Trial [27] did not find any difference in the QOL between those receiving ICD shocks, the main issue regarding quality of life is the occurrence of shocks. The PainFREE Rx II Trial [33] demonstrates very clearly that QOL can be improved by programming of antitachycardia pacing even in patients with fast VTs. This trial also documented the efficacy of antitachycardia pacing.

With regard to a potential deterioration of perceived QOL caused by delivery of ICD shocks for fast VTs, we would recommend to program ATP attempts in all ICD patients regardless of the cycle length of the index arrhythmia.

## Figures and Tables

**Table 1:**
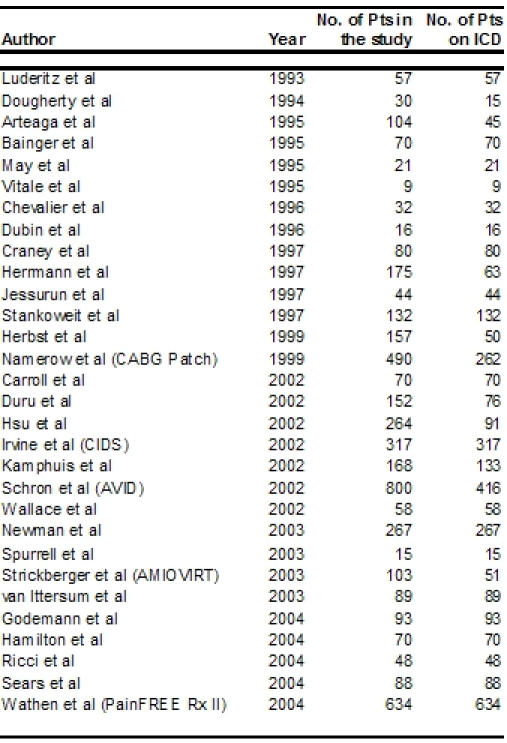
ICD Studies reporting Quality of Life Data

**Table 2:**
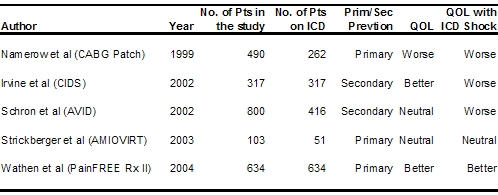
Randomized ICD  Trials Reporting Quality of Life

**Table 3:**
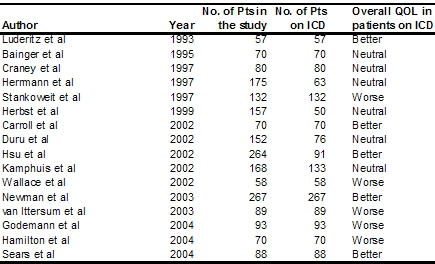
Quality of Life in Major Non Randomised Studies
